# Non-betahemolytic streptococcal bacteremia, cardiac implantable electronic device, endocarditis, extraction, and outcome; a population-based retrospective cohort study

**DOI:** 10.1007/s15010-024-02221-0

**Published:** 2024-04-18

**Authors:** Andreas Berge, Johannes Lundin, Anna Bläckberg, Torgny Sunnerhagen, Magnus Rasmussen

**Affiliations:** 1 Unit of Infectious Diseases, Department of Medicine, Solna, Karolinska Institutet, 171 76 Stockholm, Sweden; 2 Department of Infectious Diseases, Karolinska University Hospital, 171 76 Stockholm, Sweden; 3 Department of Clinical Sciences Lund, Division of Infection Medicine, Lund University, 221 00 Lund, Sweden; 4 Division for Infectious Diseases, Skåne University Hospital, 221 00 Lund, Sweden; 5 Department of Clinical Microbiology, Infection Control and Prevention, Office for Medical Services, Region Skåne, 221 85 Lund, Sweden

**Keywords:** Non-beta-hemolytic *Streptococcus*, Bacteremia, Endocarditis, CIED, Extraction, Recurrent infection

## Abstract

**Purpose:**

Patients with non-beta-hemolytic streptococcal bacteremia (NBHSB) are at risk of infective endocarditis (IE). Patients with cardiac implantable electronic device (CIED) have been described to have an increased risk of IE. The aim of the study was to describe a population-based cohort of patients with NBHSB and CIED and variables associated with IE and recurrent NBHSB.

**Methods:**

All episodes with NBHSB in blood culture from 2015 to 2018 in a population of 1.3 million inhabitants were collected from the Clinical Microbiology Laboratory, Lund, Sweden. Through medical records, patients with CIED during NBHSB were identified and clinical data were collected. Patients were followed 365 days after NBHSB.

**Results:**

Eighty-five episodes in 79 patients with CIED and NBHSB constituted the cohort. Eight patients (10%) were diagnosed with definite IE during the first episode, five of whom also had heart valve prosthesis (HVP). In 39 patients (49%) transesophageal echocardiography (TEE) was performed of which six indicated IE. Four patients had the CIED extracted. Twenty-four patients did not survive (30%) the study period. Four patients had a recurrent infection with NBHSB with the same species, three of whom had HVP and had been evaluated with TEE with a negative result during the first episode and diagnosed with IE during the recurrency.

**Conclusion:**

The study did not find a high risk of IE in patients with NBHSB and CIED. Most cases of IE were in conjunction with a simultaneous HVP. A management algorithm is suggested.

**Supplementary Information:**

The online version contains supplementary material available at 10.1007/s15010-024-02221-0.

## Introduction

Infective endocarditis (IE) is a life-threatening infection, demanding long antibiotic treatment and, under some circumstances, thoracic surgery or other interventions [1]. IE can be caused by a plethora of different bacteria [1]. The most common causative agents are *Staphylococcus aureus, Enterococcus faecalis,* and non-beta hemolytic streptococci (NBHS), together constituting the responsible agent for approximately 70% of the IE cases diagnosed [2–4]. In clinical practice, a positive blood culture (BC) with any of these bacteria, or other bacteria prone to cause IE, is often the first observation indicating that a patient has IE.

Cardiac implantable electronic devices (CIEDs) are increasingly used to treat various conditions [5]. In the circumstances with bacteremia in a patient with a CIED, an increased risk of IE has been described and extraction of the CIED has been associated with a reduced risk of treatment failure, why extraction is recommended in cases of CIED IE [6]. However, these recommendations are derived from studies of *S. aureus* IE or from tertiary referral centers [7, 8]. In one study of NBHS bacteremia (NBHSB), CIED was not found to be significantly associated to IE, and thus not included in the HANDOC score, designed to assess the risk of IE in patients with NBHSB [9]. In a large study of streptococcal blood stream infections by Chamat-Hedemand et al*.*, CIED was found to be a significant risk factor for IE (odds ratio 1.7) [10], but the exact risk of IE in the group of patients with CIED and NBHSB was not addressed. In a recent publication on NBHSB and risk factors for IE, CIED was significantly associated with IE in univariable analysis but in multivariable analysis, no correlation was seen, likely due to the covariation with other risk factors [11].

The risk of IE has been shown to be very different between different NBHS groups and species [9, 10]. However, the nomenclature and taxonomy of the NBHS are complicated and misunderstandings are common, why a comparison of the results of different studies has been impaired.

The aims of this study were to describe a population-based cohort of patients with CIED and NBHSB, the rate of IE and recurrent infection, identify variables associated to IE, and to describe the clinical presentation of the recurrent infections. We also aimed to suggest how this complicated clinical situation can be managed.

## Materials and methods

### The cohort

Information on all consecutive BCs positive for NBHS from January 2015 to December 2018, was obtained from the laboratory databases of Clinical Microbiology, Region Skåne, Lund, Sweden, the only laboratory in the region, with a catchment area population of 1.36 million inhabitants in 2018. All medical records of patients older than 18 years were studied retrospectively and patients with CIED at place at the time of the bacteremia constituted the study cohort. From these patients, detailed information was collected and stored in accordance with the ethical approval obtained from the Swedish Ethics Committee (2020-00314). Data were collected by JL and were validated by MR and AB.

### Definitions

The definitions of IE and CIED infection were used according to the European Society of Cardiology (ESC) criteria by Habib et al*.* [12]. In the last analysis in the result section, the European Heart Rhythm Association (EHRA) diagnostic criteria from Blomström-Lundqvist et al. [6], ESC 2023 guidelines diagnostic criteria [1], and the Duke-International Society for Cardiovascular Infectious Diseases (ISCVID) criteria [13]. The minor criterium predisposition to IE was used according to Dajani et al*.* [14], and in the comparison of the different diagnostic criteria systems, with the modifications described in the respective system. All changes seen on TTE or TEE, indicating IE, were considered to be caused by infection due to the difficulty to differentiate from changes due to other causes [15] and the assumed high pretest probability for CIED IE in this cohort. All infections fulfilling the criteria for definite IE were referred to as CIED IE irrespective whether changes were found on the CIED or heart valves [6].

An episode of NBHSB was defined by the start of the clinical symptoms or signs in a patient resulting in BC being taken, showing growth of NBHS. An episode was delimited by at least 7 days of effective treatment and clinical improvement or, if not fulfilled, after 30 days. A later clinical condition resulting in a BC being taken with growth of NBHS, of the same species as in the first episode, within the study period of 365 days, was referred to as a “recurrent infection” or “recurrence” and was not included in the study as a first or primary episode in Tables 1, 2, and 3. The expression “recurrent infection” or “recurrence” was used in this study since it cannot be determined whether the infection was caused by the same bacterium, indicating relapse, or by another NBHS clone indicating a reinfection.

**Table 1:** Background and presentation of the patients with CIED and NBHSB

Characteristics	All (*n* = 79) (%)	Episodes with definite IE (*n* = 8) (%)	Episodes without definite IE (*n* = 71) (%)	*p*-Value
Age (years)	84 (76–88)	84 (70–87)	84 (76–88)	0.81
Sex (female)	18 (23)	2 (25)	16 (23)	1.00
Charlson score	2 (1–4)	2 (1–4)	2 (1–4)	0.81
Acquisition				**0.046**
Community	40 (51)	7 (88)	33 (46)	0.06
Health care associated	27 (34)	1 (12)	26 (37)	0.25
Nosocomial	12 (17)	0 (0)	12 (15)	0.35
CIED implantation (years)	5 (3–8)	6 (4–8)	5 (2–8)	0.27
Type of CIED				1.00
PPM	69 (87)	7 (88)	62 (87)	
ICD	10 (13)	1 (12)	9 (13)	
Predisposition, any	24 (31)	6 (75)	18 (26)	**0.01**
Cardiac predisposition	24 (19)	6 (75)	18 (26)	**0.01**
Native valve disease	9 (11)	1 (12)	8 (11)	1.00
Prosthetic heart valve	15 (14)	5 (62)	10 (14)	**0.005**
Previous endocarditis	2 (3)	0 (0)	2 (3)	1.00
Intravenous drug user	0 (0)	0 (0)	0 (0)	1.00
Heart murmur or valve disease	27 (34)	6 (75)	21 (27)	0.97
Fever ≥ 38°	62 (78)	5 (62)	57 (80)	0.36
Embolization	1 (1)	0 (0)	1 (1)	1.00
BC major criterion for IE	42 (53)	8 (100)	34 (48)	**0.006**
Time to positive BC (h)	18 (11–29)	17 (13–22)	18 (11–30)	0.55
SOFA score (≥ 2 points)	32 (41)	4 (50)	28 (39)	0.71
Known origin of infection	22 (28)	1 (12)	21 (30)	0.67
Pocket infection	1 (1)	0 (0)	1 (1)	
Other origin of infections^a^	21 (27)	0 (0)	21 (30)	
Unknown origin of infection	57 (72)	7 (88)	50 (70)	0.43
Duration of symptoms (days)	3 (1–6)	4 (2–22)	2 (1–6)	0.13
HANDOC score (points)	2 (1–4)	5 (4–5)	1 (2–4)	** < 0.001**
Positive HANDOC score (≥ 3 points)	34 (43)	8 (100)	26 (37)	**0.001**

Univariable analysis of differences between patients diagnosed with definite IE and patients without definite IE. Values are given as numbers and proportions (%) and for continuous variables as medians and IQR. The *p*-value of differences in continuous variable were calculated with Wilcoxon’s rank sum test. In categorical variables, the differences were calculated using the χ^2^ test when applicable and Fisher’s exact test in other cases. Differences with a *p*-value of < 0.05 are considered significant and are shown in bold

^a^Most common focal infections were: abdominal focus: 10 patients, pneumonia: three patients, urinary tract infection: two patients, and oral infection: two patients

**Table 2:** BC results from the cohort of patients with CIED and NBHSB

BC results	All (*n* = 79) (%)	Episodes with definite IE (*n* = 8) (%)	Episodes without definite IE (*n* = 71) (%)	*P*-value
All NBHS BC results:				**0.047**
*S. mitis* group	29 (37)	3 (38)	26 (37)	1.00
*S. sanguinis* group	2 (3)	0 (0)	2 (3)	1.00
*S. bovis* group	17 (22)	2 (25)	15 (21)	1.00
*S. anginosus* group	16 (20)	0 (0)	16 (23)	0.20
*S. mutans* group	3 (4)	2 (25)	1 (1)	**0.03**
*S. salivarius* group	11 (14)	1 (12)	10 (14)	1.00
Other NBHS	1 (1)	0 (0)	1 (1)	1.00

Univariable logistic regression of differences between patients diagnosed with definite IE and patients without definite IE. Values are given as numbers and proportions (%). The *p*-value of differences were calculated using the χ^2^ test when applicable and Fisher’s exact test in other cases. Differences with a *p*-value of < 0.05 are considered significant and are shown in bold

**Table 3:** Management and outcome of the patients with CIED and NBHSB

Characteristics	All (*n* = 79) (%)	Episodes with definite IE (*n* = 8) (%)	Episodes without definite IE (*n* = 71) (%)	*p*-Value
*Management*				
TTE performed	54 (68)	8 (100)	46 (65)	0.051
Positive for IE	4 (5)	3 (38)	1 (1)	
TOE performed	39 (49)	7 (88)	32 (45)	**0.029**
Positive for IE	8 (10)	6 (75)	2 (3)	** < 0.001**
CIED changes	5 (6)	3 (38)	2 (3)	**0.006**
PET-CT performed	4 (5)	1 (12)	3 (4)	0.35
Positive for IE	1 (1)	1 (12)	0 (0)	0.10
CIED changes	0 (0)	0 (0)	0 (0)	1.00
Extraction of CIED	4 (3)	2 (25)	2 (3)	**0.049**
Treatment, total, (days)	13 (10–21)	28 (22–29)	13 (10–18)	** < 0.001**
*Outcome*				
Recurrence in NBHSB	4 (5)	0 (0)	4 (6)	1.0
Diagnosed with IE	3 (4)	0 (0)	3 (75)	1.0
Death within 30 days	14 (18)	1 (12)	13 (18)	1.00
Death within 365 days	24 (30)	3 (38)	21 (30)	0.69

Univariable analysis of differences between patients diagnosed with definite IE and patients without definite IE. Values are given as numbers and proportions (%) and for continuous variables as medians and IQR. The *p*-value of differences in continuous variable were calculated with Wilcoxon’s rank sum test. In categorical variables, the differences were calculated using the χ^2^ test when applicable and Fisher’s exact test in other cases. Differences with a *p*-value of < 0.05 are considered significant and are shown in bold

The primary endpoint was definite IE. The secondary endpoint was recurrent infection with the same species of NBHS during the observation period.

Origin of infection and other focal infections caused by NBHS were defined as described [16]. Comorbidities were retrieved from registrations in the medical records prior to the episode and classified according to the Charlson index modified by Quan et al*.* [17, 18]. The HANDOC scores were calculated as described [9].

### Microbiology

During the study period guidelines recommended that two sets of blood culture bottles (aerobic and anaerobic) should be taken from two separate venipunctures. The BC system in use was BACTEC FX (BectonDickinson, Franklin Lakes, United States), using a 5-day incubation unless otherwise requested. The main method for species determination was matrix-assisted laser desorption/ionization time-of-flight mass spectrometry (MALDI-TOF MS: Bruker Daltonics, using the Bruker MBT Compass library version most recent at the time of sample analysis), with a PCR and 16S sequencing using the Sanger method as a second line method for selected hard-to-identify isolates. Species designations were used according to the 2022 International Code of Nomenclature of Prokaryotes (ICPN) and the List of Prokaryotic names with Standing in Nomenclature (LPSN) [19, 20]. The grouping of streptococcal species into the *S. mitis* group, *S. anginosus* group, *S. sanguinis* group, *S. salivarius* group, *S. mutans* group, and *S. bovis* group was done in concordance with previous publications [9, 21–23].

### Data collection and analysis

The collection of the microbiological and clinical data of an episode was from 365 days before its start until 365 days after the first positive BC during that episode. The collected variables are listed in the Supplementary material. The number of CIED carriers in the Region was taken from the Swedish Pacemaker and Implantable Cardioverter-Defibrillator Registry.

The analysis of the collected data was conducted in Stata, version 15.1 (StataCorp, College Station, TX, USA). To describe the differences in dichotomous variables the χ^2^ test was used and if the prerequisites were not met, the *p-*value of Fisher’s exact test was used. Differences between continuous variables were analyzed with Wilcoxon’s rank-sum test as normal distribution was not assumed. Values are presented as proportions or medians with interquartile ranges (IQR).

## Results

### The cohort

**Fig. 1:**
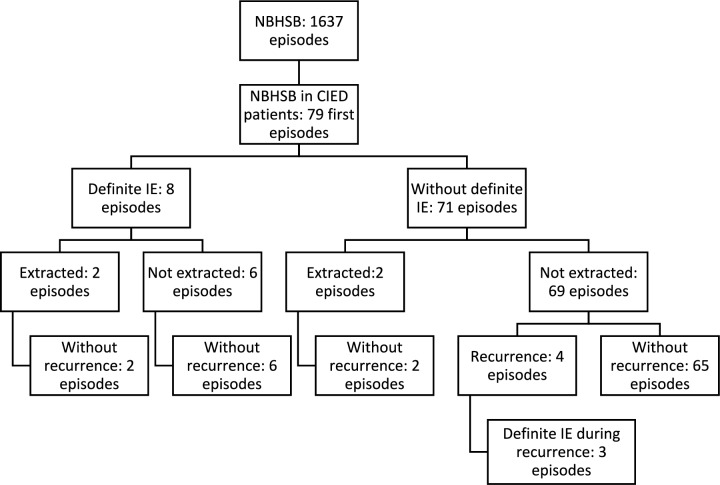
Flow chart describing the cohort of patients with CIED and NBHSB, IE, extraction of the CIED, and recurrent infection

The data extraction from the laboratory resulted in 1637 episodes of NBHSB in the four-year study period (2015–2018). In 79 patients with 85 episodes, a CIED was found at place at the time for the bacteremia. During the study period, the average number of persons with CIED and population in the region, was 8869 and 1 315,000, respectively, giving an incidence of 0.22 NBHSB/1000 CIED/year. The first episode in each patient was further studied and accounted for in Tables 1, 2, and 3. The six episodes of recurrent infections are described in Table 4. Definite IE was diagnosed during eight of the primary episodes and the CIED was extracted during four episodes. After the IE episodes, no recurrent infections were diagnosed during the observation time. The 71 episodes not diagnosed with definite IE, were followed by a recurrent episode in four patients, and IE was diagnosed in three of these patients during the recurrences (Fig. 1).

**Table 4:** The clinical presentation of the patients with recurrent episodes of NBHSB

Patient	Age	Gender	Heart valve prosthesis	NBHS group	Focus in first episode	HANDOC score/positive or negative	TOE done/result	PET-CT	Extraction during first episode	Treatment time (days)	Time EoAT^a^ to next episode (days)	Focus in second episode	TOE done/result	PET-CT done/result	Extraction of the CIED/heart operation	Treatment time	Deceased (days after last episode)
1	94	M	No	*S. mitis*	U	4/ +	No	No	No	2	40	U	No	No	No	13	No
2	82	F	Yes	*S. bovis*	U	5/ +	Yes/ −	No	No	10	19	IE	Yes/ +	No	Yes/yes	54	No
3	61	M	Yes	*S. salivarius*	U	2/ −	Yes/ −	No	No	15	81	IE	Yes/ +	No	Yes/yes	44	134
4	81	M	Yes	*S. bovis*	U	5/ +	Yes/ −	No	No	15	20	IE^b^	Yes/ −	Yes/ +	Yes/yes	54	No

*U* unknown focus; *IE* endocarditis, + positive finding

^a^End of antibiotic therapy

^b^Three recurrent episodes, extraction of CIED and heart surgery during the third after positive PET-CT; last episode without known focus

### Variables associated with IE

For the first episodes in the patients of the cohort, clinical variables are presented using univariable analysis to identify variables associated with IE (Table 1). Community acquisition was more common in the IE group. Predisposition and one of its components, HVP, and a BC fulfilling the major criterion for IE were significantly more common among patients diagnosed with IE. Both the HANDOC score as a continuous variable and the positive HANDOC score with the stipulated cut off of ≥ 3 points were significantly associated with IE.

In Table 2, the distribution of IE and non-IE episodes, between the different groups of species constituting the NBHS are described. The patients with bacteremia due to the *S. mutans* group of species had two episodes of IE out of three episodes and none of the 16 episodes of the *S. anginosus* group bacteremias were diagnosed with IE. The distribution was significantly different from the one expected by chance, *p*-value 0.047 (Table 2).

### Management and outcome

The management and outcome of the patients in the 79 episodes are described (Table 3) using univariable analysis. In 56 episodes (71%) an echocardiography was performed, 39 patients (49%) were examined with TEE. Four patients were evaluated with PET-CT, out of which one was indicative of IE. No cardiac CT was performed. In 62 episodes, a total of 83 examinations (51 radiology of the lungs, 13 CT of the lungs, and 19 CT of the abdomen) were performed to identify embolic events. Of these, two indicated embolization.

The CIED was extracted in four patients, all with changes visualized on the CIED, and two fulfilling the criteria for definite IE. No patient had a positive culture from an extracted CIED and in one patient, the 16S analysis of the extracted CIED confirmed the BC result. The 1-year overall mortality in the entire cohort was 30% and the median time to death was 19 days (IQR 6–80 days). The comparison between patients diagnosed with definite IE and without the diagnosis demonstrated significant differences in the rate of echocardiography performed, the rate of extraction, and the treatment time (Table 3). No significant differences were seen in recurrences, mortality, or time to death from the positive BC. Three patients diagnosed with definite CIED IE died during the observation time, after 9, 58, and 95 days, respectively, all without extraction of the CIED. The mortality in each group of NBHS species was analyzed: in episodes caused by bacteria from the *S. sanguinis* and *S. mutans* groups none of the five patients died, while in the *S. salivarius* group 7/11 patients (64%) died during the study period. However, differences in mortality between the different bacterial groups were not statistically significant (data not shown).

### The recurrent infections

In the four episodes followed by a recurrent NBHSB, three of the patients had HVP and in the same three TEE, but not PET-CT, was performed during the first episode (Table 4). No focal infection was diagnosed in any of these four patients and the patients were treated with antibiotics for 2, 10, 15, and 15 days, respectively. The patients had positive BC with the same group of NHBS in both episodes. Two of the patients with recurrence had bacteremia with an isolate of the *S. bovis* group, one from the *S. salivarius* group, and one from the *S. mitis* group.

The three patients with HVP were all diagnosed to have an IE during the recurrency. Two of the patients had the major structural criterion identified by a positive TEE and one had a PET-CT showing IE. In none of the episodes of recurrent infections there were visible changes on the CIED. Two of these patients were subjected to surgery and one died during the treatment but 54 days after the second episode (Table 4).

### Alternative diagnostic criteria systems

In this study, the diagnostic criteria of ESC 2015 were used [12]. A comparison with three other diagnostic criteria systems is described in Table 5. The ESC 2015 and EHRA 2020 result in the same distribution of definite, possible, and rejected IE. The results of the ESC 2023 and the Duke-ISCVID diagnostic criteria systems result in the same distribution of classification of IE but differs from the former, as one additional episode was classified as definite IE and four episodes were reclassified from rejected IE to possible IE (Table 5).

**Table 5:** The outcome of four different diagnostic criteria systems

Patients, *n* = 79	ESC 2015	EHRA 2020	ESC 2023	Duke-ISCVID 2023
Definite IE	8	8	9	9
Possible IE	39	39	42	42
Rejected IE	33	33	28	28

The diagnostic criteria systems have been abbreviated: ESC 2015 [12], EHRA 2020 [6], Duke-ISCVID 2023 [13], and the ESC 2023 [1]. The ESC 2015 diagnostic criteria were used in this study

## Discussion

The main finding in this study is that IE is found in 10% of a population-based cohort of patients with CIED and NBHSB and extraction of the CIED was not performed unconditionally in cases of CIED IE. Further, a low rate of recurrent infections (5%) was encountered. Finally, three patients were diagnosed with IE during the recurrent infections, all related to an HVP.

The role of CIED in the pathogenesis of IE is debated. Whether CIED is a risk factor for IE, most likely depends on the species of the causing bacterium. In studies of patients with *S. aureus* bacteremia it was demonstrated that CIED was a risk factor for IE [7, 24] while in *Enterococcus faecalis* bacteremia, the presence of a CIED was not shown to be associated with IE [16]. In a previous study of NBHSB [9], CIED was not significantly associated with IE (but with a *p*-value close to 0.05). In that study, CIED covaried with HVP, which was shown to predict IE (unpublished data). In another NBHSB and IE study, CIED was associated to IE in univariable analysis but in multivariable analysis, an odds ratio of 0.66 was found [11]. Chamat-Hedemand et al*.* described a large cohort of patients with patients with CIED and streptococcal bacteremia but in the calculation resulting in CIED being a significant risk factor for IE, the patients with bacteremia with species addressed in this study only constitute a minority (approximately 30%).

In two alternative IE diagnostic criteria systems [1, 13], CIED is included among the different conditions constituting the predisposition minor criterium, resulting in that all patients in this cohort have that minor criterion. This change in the diagnostic criteria did not result in any profound differences in the rates of definite and possible IE. The data presented in this study neither indicate CIED to be a strong risk factor for IE in NBHSB nor that it contributes to a better performance for the diagnostic criteria if introduced as a minor criterion.

The results of this study were in line with the findings in several studies [9, 21, 25], showing that the risk of IE was diverse between groups of NBHS. *S. sanguinis, S. bovis,* and *S. mutans* are prone to cause IE, *S. mitis* and *S. salivarius* are connected to an intermediate risk, and *S. anginosus* is unlikely to cause IE.

In only four patients (5%) in the entire cohort, the CIED was extracted and in patients fulfilling the criteria for definite IE, two out of 8 (25%) of the patients had the CIED extracted. None of the patients with IE had a recurrent infection that would indicate treatment failure. The guidelines recommend extraction of the CIED in cases diagnosed with CIED infection or CIED IE [6] but this was not performed in our cohort, challenging the necessity to follow the recommendations.

Four out of 71 patients (6%) not diagnosed with IE had a recurrent infection with NBHSB, and none was found to have CIED changes. However, three patients had suspected undiagnosed left sided HVP IE. Although beyond the scope of this study, the three missed HVP IE with recurrent infections illustrate the importance of continuing the evaluation of a patient with HVP, NBHSB, and a negative TEE. Such patients could be subjected to PET-CT, cardiac CT, repeated TEE, or followed clinically for early detection of a relapse [6, 12, 13].

**Fig. 2:**
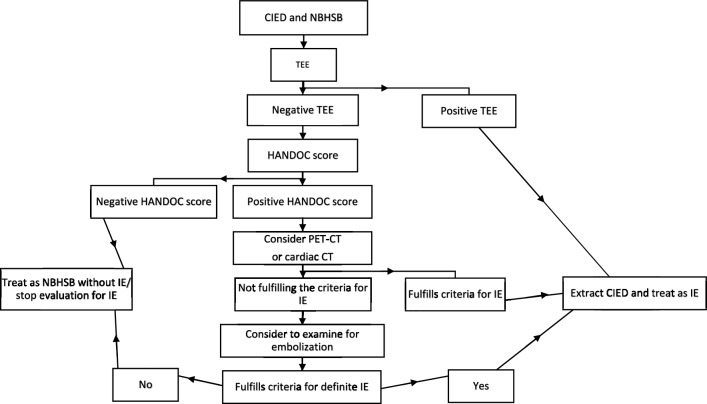
Flow chart for the suggested management strategy for patients with CIED and NBHSB

Based on our results, we suggest a management strategy that includes that all patients should be evaluated with TEE and, if negative, a PET-CT or possibly cardiac CT could be considered if the suspicion of CIED IE remains. The size of our study does not permit us to identify specific risk factors for IE in patients with CIED and thus the HANDOC score is suggested to be used to direct the management after a negative TEE. None of the diagnosed episodes of definite IE, nor any of the patients with a recurrent infection, had a negative HANDOC-score. Thus, we propose that the risk of IE would be negligible with both a negative TEE and a negative HANDOC score and further evaluation for IE could be omitted (Fig. 2). Another line of inquiry would be to test the hypothesis that TEE can be omitted in patients with CIED, NBHSB, and a negative HANDOC score. However, this suggestion has to be tested in future prospective studies.

Although this is the largest study cohort focusing on CIED-carrying patients with NBHSB, it has obvious limitations. The retrospective design and the far from complete evaluation using TEE (49%), make it possible that some patients with changes on the CIED could have been missed. Moreover, only four patients were subjected to PET-CT, also possibly contributing to under-diagnosis. Furthermore, despite the long follow-up and thorough evaluation of the medical records, some patients could have died of an undiagnosed IE, another undiagnosed NBHS infection, or a recurrent infection. Finally, we do not know if the recurrent infections were true relapses or reinfections with another clone from the same group NBHS.

Despite the shortcomings, we believe that the observation of low frequency of CIED infections in NBHSB and the suggestion of a management algorithm has implications for the management of the patients.

## Supplementary Information

Below is the link to the electronic supplementary material.

Supplementary file1 (DOCX 27 KB)

## Data Availability

The datasets analyzed during the current study are available from the corresponding author on reasonable request.
