# Supplementary material for: Non-betahemolytic streptococcal bacteremia, cardiac implantable electronic device, endocarditis, extraction, and outcome; a population-based retrospective cohort study
Source: Infection. 2024 Apr 18;52(5):1911–9. doi: 10.1007/s15010-024-02221-0 (PMC11499341; doi:10.1007/s15010-024-02221-0)
Supplement: Supplementary file 1 — Supplementary file1 (DOCX 27 KB) [file 15010_2024_2221_MOESM1_ESM.docx]

Supplementary material

*Data collection*

Data were collected from 365 days before the BC defining an episode and 365 days after the day of the BC. The number of BC taken and the number of positive BC, whether BC were taken after the start of adequate therapy and the time to the last positive BC, culture results other than BC taken during the episode, the result of other microbiological tests (including 16S analysis from extracted CIED material, cultures and other analyses) were collected. Data were collected on age, gender, acquisition of the infection, community, health care associated, and nosocomial, according to Friedman *et al.* (26), comorbidities according to modified Charlson score (18), the existence of and duration of symptoms and signs of local and systemic infection during the episode, and performed radiology and its results. Day of death after positive BC within 365 days, days hospitalized were registered. Further, cultures or clinical conditions indicating therapeutic failure during follow up were registered, both for patients surviving the study period and the patients that died during the study period. Further, data were collected on heart murmur, intravenous drug use, predisposing heart conditions, fever, vascular or immunological phenomena, microbiological data fulfilling or not fulfilling the prerequisites for Duke minor or major criteria (27). Also, data were collected from whether TTE, TEE, PET-CT, and cardiac CT were performed and the findings and if the findings were restricted to the CIED or also or exclusively on other structures constituting the structural major criterion of IE (6, 27). The data were compiled to identify if the diagnostic criteria for possible and definite IE were met (6). Further, data on the CIED and its history were collected: type of CIED, date of implantation and revision in relation to the episode, previous CIED and the reason for its removal. Extraction decision and its result, extraction performed, and time from BC during the episode or within the remaining observation period of 365 days was noted separately to distinguish extraction as a primary management measure in contrast to extraction due to recurrent infection or other causes. Data on choice of treatment duration, group of antibiotics, changes during treatment, combination of antibiotics, and intravenous or oral treatment were collected. Treatment with intensive care and thoracic surgery was noted.

Missing data were registered as lack of result in that variable. No imputations were made.

Microbiology

The BACTEC FX BC system (Becton Dickinson, Franklin Lakes, NJ, USA) was used with the BD BACTEC culture media (Plus Aerobic/F, Lytic/10 Anaerobic and Peds Plus/F). Species determination was performed with Microflex MALDI-TOF mass spectrometry (MS), using the direct transfer method (28). The generated mass spectra of the bacterial isolates were analyzed with the MALDI Biotyper 4.1 software and the MALDI Biotyper Library DB-8468 (Bruker, Bremen, Germany). Identification of growth of bacteria from pocket tissue biopsy and extracted CIED was done according to standard laboratory procedures, CIED leads were analyzed by sequencing the 16S rRNA gene and assigned a species (29).
